# Quantitative distribution of iodinated contrast media in body computed tomography: data from a large reference cohort

**DOI:** 10.1007/s00330-020-07298-3

**Published:** 2020-09-30

**Authors:** David Zopfs, Josefine Graffe, Robert Peter Reimer, Sebastian Schäfer, Thorsten Persigehl, David Maintz, Jan Borggrefe, Stefan Haneder, Simon Lennartz, Nils Große Hokamp

**Affiliations:** 1 University Cologne, Faculty of Medicine and University Hospital Cologne, Department of Diagnostic and Interventional Radiology, Cologne, Germany; 2 Faculty of Medicine, University of Cologne, Cologne, Germany; 3 Mint Medical GmbH, Heidelberg, Germany; 4 Department of Radiology, Massachusetts General Hospital, Harvard Medical School, Boston, MA USA

**Keywords:** Contrast media, Reference values, Tomography, X-ray computed, Tumor burden, Biomarkers, tumor

## Abstract

**Objectives:**

Dual-energy computed tomography allows for an accurate and reliable quantification of iodine. However, data on physiological distribution of iodine concentration (IC) is still sparse. This study aims to establish guidance for IC in abdominal organs and important anatomical landmarks using a large cohort of individuals without radiological tumor burden.

**Methods:**

Five hundred seventy-one oncologic, portal venous phase dual-layer spectral detector CT studies of the chest and abdomen without tumor burden at time point of imaging confirmed by > 3-month follow-up were included. ROI were placed in parenchymatous organs (*n* = 25), lymph nodes (*n* = 6), and vessels (*n* = 3) with a minimum of two measurements per landmark. ROI were placed on conventional images and pasted to iodine maps to retrieve absolute IC. Normalization to the abdominal aorta was conducted to obtain iodine perfusion ratios. Bivariate regression analysis, *t* tests, and ANOVA with Tukey-Kramer post hoc test were used for statistical analysis.

**Results:**

Absolute IC showed a broad scatter and varied with body mass index, between different age groups and between the sexes in parenchymatous organs, lymph nodes, and vessels (range 0.0 ± 0.0 mg/ml–6.6 ± 1.3 mg/ml). Unlike absolute IC, iodine perfusion ratios did not show dependency on body mass index; however, significant differences between the sexes and age groups persisted, showing a tendency towards decreased perfusion ratios in elderly patients (e.g., liver 18–44 years/≥ 64 years: 0.50 ± 0.11/0.43 ± 0.10, *p* ≤ 0.05).

**Conclusions:**

Distribution of IC obtained from a large-scale cohort is provided. As significant differences between sexes and age groups were found, this should be taken into account when obtaining quantitative iodine concentrations and applying iodine thresholds.

**Key Points:**

• *Absolute iodine concentration showed a broad variation and differed between body mass index, age groups, and between the sexes in parenchymatous organs, lymph nodes, and vessels.*

• *The iodine perfusion ratios did not show dependency on body mass index while significant differences between sexes and age groups persisted.*

• *Provided guidance values may serve as reference when aiming to differentiate healthy and abnormal tissue based on iodine perfusion ratios.*

## Introduction

Computed tomography is the modality of choice for body imaging in oncologic patients. It allows for a fast and inexpensive visualization of tumor burden at initial staging as well as in follow-up examinations and is therefore essential to warrant stage-adapted treatment. To assess this quantitatively, different reporting criteria have been established; most of these rely on mono- or bidimensional measurements of morphologic tumor size (morphometric methods). Among these, the response evaluation criteria in solid tumors (RECIST) are most frequently used [1–4]. For some tumors, additional efforts have been made in order to incorporate functional information to some extent. For example, in staging of hepatocellular carcinoma, reporting criteria have been suggested (i.e., LI-RADS) that suggest to include visually viable tumor tissue, only [5, 6].

In oncologic computed tomography imaging, iodinated contrast media are almost always administered in order to overcome the low intrinsic soft tissue contrast and enable a sufficient delineation of vessels, organs, and lymph nodes. Furthermore, tumor vascularization is an important diagnostic aspect in assessment of treatment response in some tumors [7–9]. Dual-energy computed tomography (DECT) allows for an accurate and reliable quantification of iodine and therefore provides information regarding the distribution of iodinated contrast media [10–15]. Accuracy of iodine quantification has been demonstrated for all different technical approaches to DECT [12, 16, 17]. Results of iodine quantification are commonly presented as gray-scaled images referred to as iodine maps (IM). Despite meeting conformity with the digital imaging and communications in medicine (DICOM) standard, pixel values of these images represent iodine concentration as mass per volume (commonly milligram per milliliter [mg/ml]) as opposed to CT attenuation in Hounsfield units (HU).

Being a quantitative parameter obtainable with high accuracy [14, 15, 18], iodine concentration can be used as a surrogate parameter for perfusion of any given organ/structure at the time point of image acquisition [19]. Various studies have already proposed quantitative iodine values to differentiate between malignant and healthy tissue or used quantitative iodine to predict treatment response [20–25]. To pave the road towards clinical application of iodine quantification, which is particularly of interest for oncologic imaging, it is pivotal to understand normal distribution and cohort-specific variance of these values. However, data on physiological iodine distribution in subjects without tumor burden is sparse; only few studies report reference values for lymph nodes and myocardial tissue [26, 27]. This study aims to establish aims to establish guidance values for absolute iodine concentrations and perfusion ratios of abdominal organs, lymph nodes, and other important anatomical landmarks using a large cohort of individuals without radiological tumor burden.

## Materials and methods

### Patient acquisition

The institutional review board approved this monocentric, retrospective study waiving the requirement for informed consent (Ref-No. 18-171). A combined query to the radiological information system and the picture archiving and communication system was performed for patients fulfilling following criteria:

I. ≥ 18 years;
II. Contrast-enhanced, portal venous phase thoracoabdominal staging CT from 5/2016 to 12/2019 on a dual-layer spectral detector computed tomography (SDCT) scanner;
III. Diagnosed dermato-oncological disease (malignant melanoma, squamous cell carcinoma, primary cutaneous B cell lymphoma, or Merkel cell carcinoma);
IV. No tumor burden confirmed by follow-up imaging after a period of at least 3 months by a board-certified radiologist.

Examinations of patients with additional secondary malignancies and active tumor burden were excluded (*n* = 920). Furthermore, examinations with deviation from the standardized examination protocol (*n* = 7) and missing follow-up data (*n* = 23) were excluded. Additionally, during quantitative assessment, all scans were screened for radiologically assessable conditions that may impact measurement accuracy; however, no patients were excluded based on this procedure. Patients’ body mass index (BMI, [kg/m^2^]) at the time of the examination was recorded and three groups were defined with regard to age: (I) 18–44 years, (II) 45–64 years, and (III) ≥ 65 years.

### Imaging protocol

All patients underwent clinically indicated thoracoabdominal spectral detector CT (IQon, Philips Healthcare) for oncologic staging. Every patient was scanned in supine position. The standardized study protocol consisted of a portal venous phase acquisition with following scan settings: tube voltage 120 kVp, tube current modulation (DoseRight 3D-DOM, Philips Healthcare), rotation time 0.33 s, pitch 0.671, collimation 64 × 0.625 mm, matrix 512 × 512, default reconstruction field-of-view was (500 × 500) × mm^2^ , resulting in a CDTI_vol_ of 11.5 mGy. A total of 100 ml of iodinated contrast media (Accupaque, 350 mg/ml; GE Healthcare) is routinely administered as a bolus using a 20-G intravenous catheter with a flow rate of 3.0 ml/s, followed by a saline flush of 30 ml. To trigger image acquisition, bolus tracking technique was used. Images were acquired with a delay of 50 s after reaching the threshold of 150 HU in the descending aorta.

### Postprocessing

For each scan, conventional images (CI) and IM were reconstructed from the same spectral dataset using the vendor’s image viewing and postprocessing tool (IntelliSpace Portal v10, Philips Healthcare). To generate CI, a hybrid-iterative reconstruction algorithm was used, and the standard soft tissue kernel was chosen (iDose^4^, denoising level 3/7, filter B, level 3, Philips Healthcare). IM were reconstructed using a dedicated, hybrid-iterative spectral reconstruction method and the same kernel (Spectral, denoising level 3/7, filter B, level 3, Philips Healthcare). A 2-mm slice thickness and a 1-mm section increment were chosen for all datasets.

### Quantitative data acquisition

**Fig. 1:**
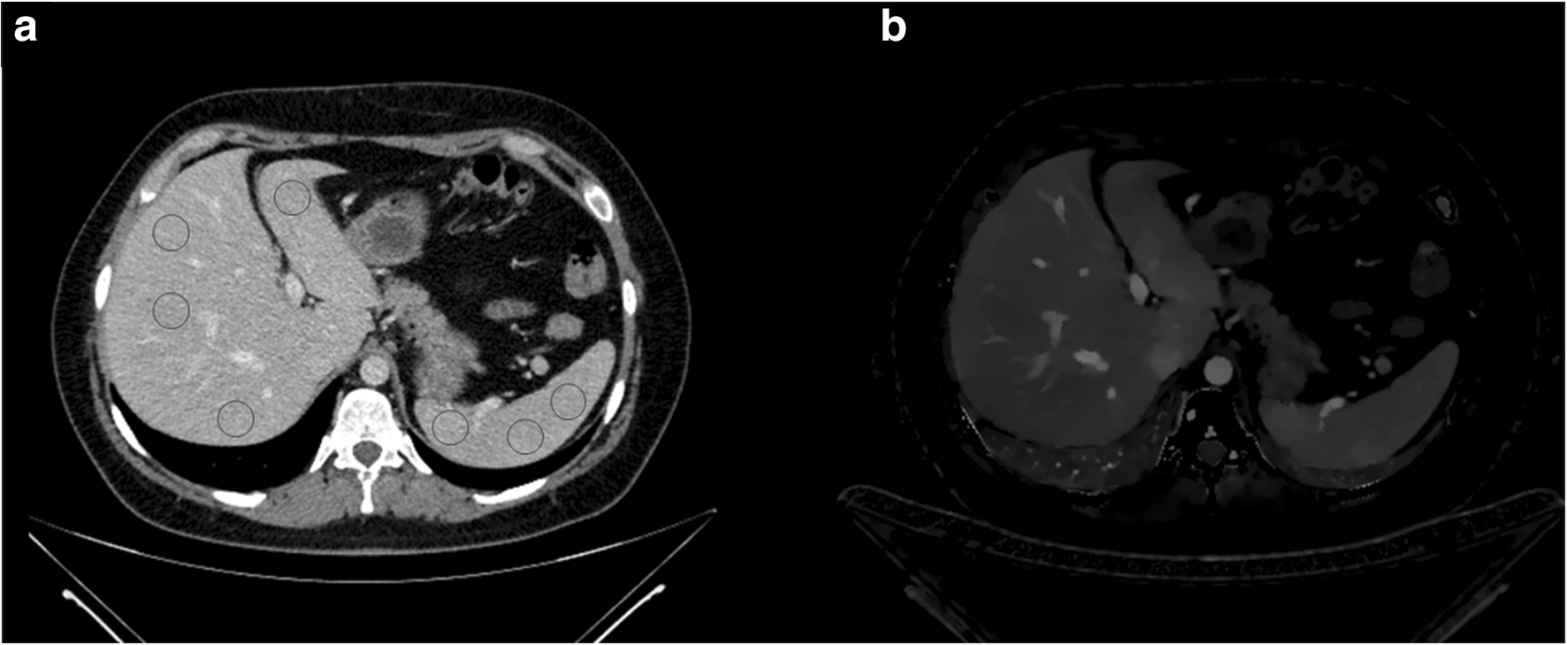
Regions-of-interest were placed in conventional images (**a**) and then copied to corresponding iodine maps (**b**)

CI and IM were transferred from the picture archiving and communication system to a proprietary software for quantitative analysis of oncologic examinations (mint lesion researchv3.6, Mint Medical GmbH). ROI were placed in CI and copied to identical positions in IM to warrant exact size and location. The target size of each ROI was 10 × 10 mm, while an inclusion of surrounding structures was avoided. A total of 37 ROI per examination were placed in organs, lymph nodes, fat, and muscle as depicted in Table 1 and Fig. 1. Particularly, measurements in the abdominal aorta were conducted to allow for data normalization resulting in the iodine perfusion ratio. For each ROI, iodine concentration and corresponding standard deviation was exported. To assess the spread of IC and iodine perfusion ratio, the so-called distribution parameter was calculated as $\mathrm{distribution}\ \mathrm{parameter}=\frac{\mathrm{IQR}}{\mathrm{R}}=\frac{x_{0.75}-{x}_{0.25}}{x_1-{x}_0}$, (IQR: interquartile range, R: range).

**Table 1:** Detailed overview of all 37 measurements. Ellipsoid region-of-interest (ROI) were placed as described above

Structure	Number of ROI	Location
Liver	9	Liver segment I–VIII
Spleen	3	Ventral, center, dorsal
Pancreas	3	Tail, body, head
Adrenal glands	2	Left and right adrenal gland
Renal cortex	6	Each side: upper pole, center, lower pole
Prostate/uterus	2	Left and right prostate lobe/fundus of uteri (2)
Psoas muscle	2	Each side at the height of the third lumbar vertebrae
Subcutaneous fat	1	Abdominal wall
Lymph nodes	6	Right axillary, left axillary, right inguinal, left inguinal, periaortic (2), and mediastinal (2)
Portal vein	1	Extrahepatic
Descending aorta	2	At the height of the tracheal bifurcation and the aortic bifurcation

### Statistical analysis

Statistical analysis was performed using JMP Software (v14, SAS Institute). Continuous variables are provided as mean ± standard deviation. Bivariate regression analysis, *t* tests, and ANOVA with Tukey-Kramer post hoc were used for statistic assessment of data. Statistical significance was defined as *p* ≤ 0.05.

## Results

### Patient collective

Patients mean age was 61.3 ± 16.8 years (range 23–93 years), 54% were female and 46% were male. Mean BMI was 27.8 ± 5.5 kg/m^2^, ranging from 16.0–51.4 kg/m^2^. At the timepoint of imaging, in 442 cases, no therapy was performed. Medication was prescribed in 129 cases, which were either checkpoint inhibitors (*n* = 68), tyrosine kinase inhibitors (*n* = 23), or interferons (*n* = 38); no patient received antiangiogenetic medication. Further characteristics are reported in detail in Table 2.

**Table 2:** Body mass index (BMI) with corresponding standard deviation and age groups for both genders

Gender	Age groups	*N*	BMI
Female	18–44 years	35	24.7 ± 5.6
	45–64 years	128	28.2 ± 6.5
	≥ 64 years	143	26.8 ± 4.2
Male	18–44 years	50	26.4 ± 4.9
	45–64 years	104	29.8 ± 6.4
	≥ 64 years	111	28.4 ± 4.4

### Absolute iodine concentrations

**Fig. 2:**
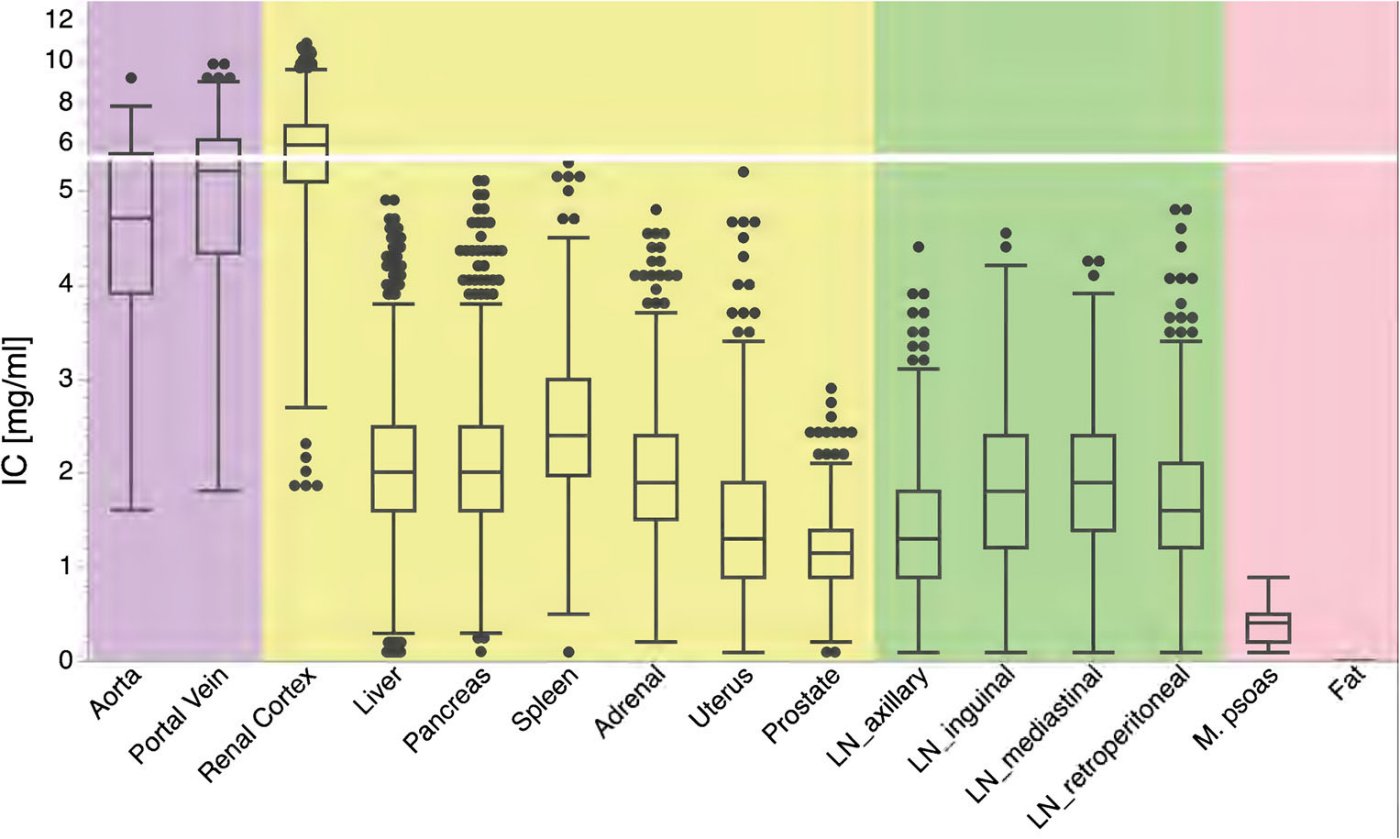
Box plots of average iodine concentration (IC) in vessels (violet), organs (yellow), lymph nodes (LN, green), as well as muscle and fat (red)

Highest and lowest iodine concentrations were found in the renal cortex (10.9 mg/ml, average 6.1 ± 1.3 mg/ml) and fat (0.0 mg/ml, average 0.0 ± 0.0 mg/ml), respectively. In parenchymatous organs (i.e., liver, pancreas, spleen, and adrenal), average iodine concentration was 2.2 ± 0.7 mg/ml ranging from 0.1 mg/ml to 5.4 mg/ml (Fig. 2). Iodine concentration in lymph nodes ranged from 0.1 to 4.8 mg/ml, exhibiting an average of 1.7 ± 0.7 mg/ml. While fat did not show any iodine uptake, mean iodine concentration in the muscle was 0.4 ± 0.2 mg/ml (range 0.1–0.9 mg/ml).

**Fig. 3:**
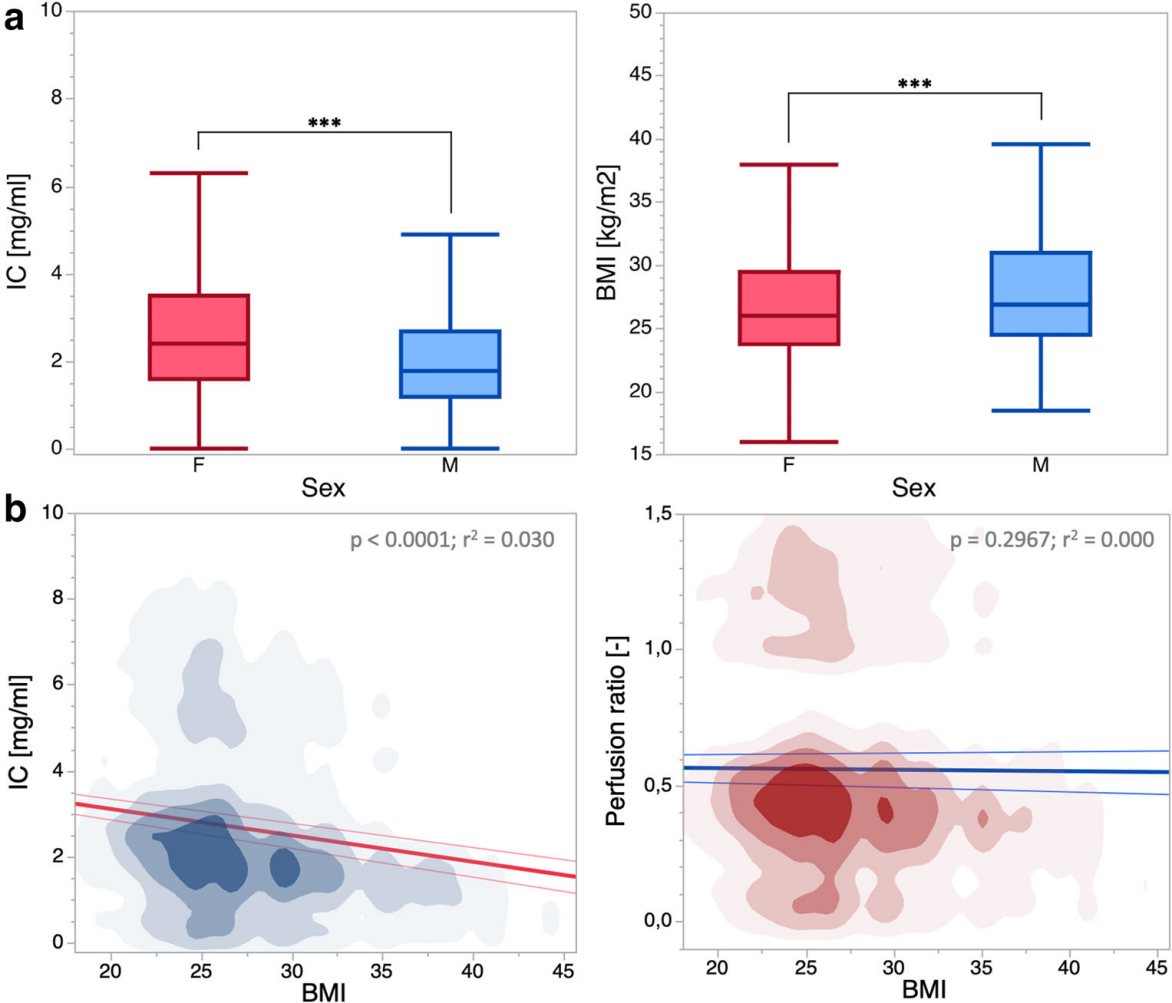
**a** Box plots of averaged iodine concentration (IC) and body mass index (BMI) in male (M) and female (F) patients. Statistical significance is indicated by an asterisk (***p > 0.001). **b** Density plots of all measurements in dependency of the body mass index (BMI) for IC and perfusion ratio. Results obtained from regression analysis are depicted in the top right corner. Notably, after normalization of IC to the aorta, BMI was not deemed a significant parameter in regression analysis

When analyzing differences in iodine concentration with regard to sex, generally higher results for female patients were observed (all *p* ≤ 0.05, Fig. 3, Table 3). However, female patients demonstrated a significantly lower BMI in comparison with male patients. Furthermore, BMI was determined a significant factor in regression analysis (*p* < 0.001, *r*^2^ = 0.03, Fig. 3). To adjust the amount of contrast media to body weight, all data was normalized to the abdominal aorta.

**Table 3:** Detailed gender-specific means of absolute iodine concentrations, age, and body mass index (BMI). Statistical significant *p* values are indicated in bold

	Female (mg/ml)	Male (mg/ml)	*p* values (M vs F)
All ROI	2.9 ± 2.1	2.3 ± 1.7	**0.001**
Aorta	5.4 ± 1.0	4.1 ± 0.9	**0.001**
Portal vein	6.0 ± 1.2	4.5 ± 1.0	**0.001**
Renal cortex	6.6 ± 1.3	5.5 ± 1.1	**0.001**
Liver	2.3 ± 0.7	1.8 ± 0.6	**0.001**
Pancreas	2.4 ± 0.7	1.7 ± 0.5	**0.001**
Spleen	2.9 ± 0.7	2.0 ± 0.6	**0.001**
Adrenal	2.2 ± 0.7	1.7 ± 0.6	**0.001**
Uterus	1.5 ± 0.9	0.0 ± 0.0	n/a
Prostate	0.0 ± 0.0	1.2 ± 0.4	n/a
Axillary lymph nodes	1.5 ± 0.8	1.2 ± 0.6	**0.001**
Inguinal lymph nodes	2.0 ± 0.9	1.6 ± 0.7	**0.001**
Mediastinal lymph nodes	1.9 ± 0.7	1.8 ± 0.7	**0.002**
Retroperitoneal lymph nodes	1.7 ± 0.7	1.6 ± 0.7	**0.004**
Psoas muscle	0.4 ± 0.2	0.3 ± 0.1	**0.001**
Fat	0.0 ± 0.0	0.0 ± 0.0	n/a
BMI	27.1 ± 5.5	28.6 ± 5.5	**0.001**
Age	61.9 ± 16.4	60.6 ± 17.2	0.181

### Normalized iodine perfusion ratios

**Fig. 4:**
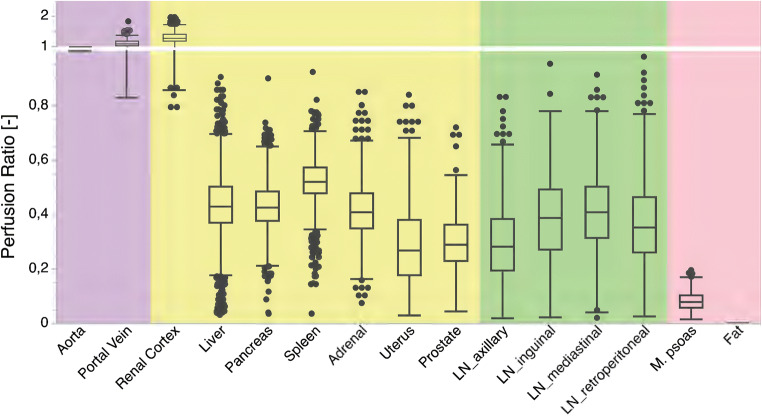
Iodine perfusion ratio in vessels (violet), organs (yellow), lymph nodes (LN, green), and muscle and fat (red)

After normalization, iodine perfusion ratio, a surrogate for organ perfusion, was obtained (Fig. 4). In contrast to absolute iodine concentration, contribution from BMI was not determined a significant predictor for iodine perfusion ratios in regression analysis (*p* = 0.30). Iodine perfusion ratios in prostate, uterus, muscle, and fat were markedly lower (0.30 ± 0.10, 0.30 ± 0.16, 0.08 ± 0.03, and 0.00 ± 0.00). Furthermore, distribution was significantly narrower for iodine perfusion ratios compared with IC (mean:0.18 and 0.22, *p* ≤ 0.05, Table 4).

**Table 4:** Distribution parameter to assess the spread of absolute iodine concentration in comparison to iodine perfusion ratio

	Iodine concentration	Iodine perfusion ratio
Aorta	n/a	n/a
Portal vein	0.25	0.15
Renal cortex	0.22	0.19
Liver	0.19	0.15
Pancreas	0.17	0.13
Spleen	0.19	0.10
Adrenal	0.20	0.17
Uterus	0.18	0.25
Prostate	0.18	0.20
Axillary lymph nodes	0.21	0.17
Inguinal lymph nodes	0.27	0.24
Mediastinal lymph nodes	0.24	0.21
Retroperitoneal lymph nodes	0.19	0.21
Psoas muscle	0.38	0.22
Fat	n/a	n/a
Mean	0.22	0.18

### Impact of sex and age on perfusion ratio

Remarkable differences between female and male patients were observed for iodine perfusion ratios of renal cortex, pancreas, spleen, and lymph nodes (Table 5, all ≤ 0.05). While renal cortex and lymph nodes demonstrated higher perfusion ratios in male patients, the other structures showed higher or comparable perfusion ratios in female patients (Table 5). Interestingly, perfusion ratio of the liver was lower in middle aged and elder patients (0.50 ± 0.11 versus 0.41 ± 0.10, 0.43 ± 0.10, *p* ≤ 0.05). In women, perfusion ratio of the uterus showed a decline with increase in age (e.g., 0.41 ± 0.16, 0.33 ± 0.17, and 0.21 ± 0.11 in 18–44, 45–64, and ≥ 64 years old women, respectively, all *p* ≤ 0.05). The prostate, on the other hand, demonstrated higher perfusion ratios in young men (i.e., 18–44 years) while perfusion ratios in middle and elder men remained constant (0.35 ± 0.11 versus 0.29 ± 0.10 and 0.28 ± 0.10, each *p* ≤ 0.05). Further detailed for differences between sex and age are presented in Table 5.

**Table 5:** Detailed iodine perfusion ratios with regard to gender and age groups. Statistical significance is indicated in italics

	Female	Male	*p* values	18–44 years	45–64 years	≥ 64 years	*p* values		
	**(-)**	**(-)**	M vs F	**(-)**	**(-)**	**(-)**	18–44 vs 45–64 years	45–64 vs ≥ 64 years	18–44 vs ≥ 64 years
Portal vein	1.11 ± 0.12	1.12 ± 0.14	0.694	1.13 ± 0.11	1.10 ± 0.11	1.12 ± 0.16	0.302	0.310	0.897
Renal cortex	1.24 ± 0.14	1.36 ± 0.18	*< 0.001*	1.35 ± 0.18	1.33 ± 0.15	1.25 ± 0.18	*< 0.001*	*< 0.001*	0.071
Liver	0.43 ± 0.09	0.43 ± 0.12	0.305	0.50 ± 0.11	0.41 ± 0.10	0.43 ± 0.10	*< 0.001*	*< 0.001*	*< 0.001*
Pancreas	0.45 ± 0.09	0.41 ± 0.10	*< 0.001*	0.47 ± 0.08	0.43 ± 0.09	0.42 ± 0.09	*< 0.001*	*< 0.001*	0.509
Spleen	0.54 ± 0.07	0.49 ± 0.10	*< 0.001*	0.53 ± 0.05	0.50 ± 0.09	0.53 ± 0.09	*0.001*	*< 0.001*	0.964
Adrenal	0.42 ± 0.10	0.41 ± 0.12	0.832	0.44 ± 0.11	0.40 ± 0.11	0.42 ± 0.11	*< 0.001*	*0.005*	0.221
Uterus	0.30 ± 0.16		n/a	0.41 ± 0.16	0.33 ± 0.17	0.21 ± 0.11	*< 0.001*	*< 0.001*	*0.004*
Prostate		0.30 ± 0.10	n/a	0.35 ± 0.11	0.29 ± 0.10	0.28 ± 0.10	*< 0.001*	*< 0.001*	0.835
Axillary lymph nodes	0.27 ± 0.13	0.31 ± 0.15	*< 0.001*	0.34 ± 0.13	0.29 ± 0.14	0.27 ± 0.14	*< 0.001*	*0.006*	0.1
Inguinal lymph nodes	0.38 ± 0.15	0.40 ± 0.16	0.203	0.48 ± 0.13	0.40 ± 0.16	0.34 ± 0.15	*< 0.001*	*< 0.001*	*< 0.001*
Mediastinal lymph nodes	0.37 ± 0.13	0.45 ± 0.15	*< 0.001*	0.34 ± 0.14	0.42 ± 0.15	0.42 ± 0.13	*< 0.001*	*< 0.001*	0.991
Retroperitoneal lymph nodes	0.33 ± 0.14	0.40 ± 0.15	*< 0.001*	0.36 ± 0.13	0.38 ± 0.15	0.35 ± 0.15	0.01	0.41	0.729
Psoas muscle	0.08 ± 0.03	0.08 ± 0.04	*< 0.001*	0.08 ± 0.03	0.08 ± 0.04	0.08 ± 0.03	0.278	0.401	0.930
Fat	0.00 ± 0.00	0.00 ± 0.00	n/a	0.00 ± 0.00	0.00 ± 0.00	0.00 ± 0.00	n/a	n/a	n/a

## Discussion

This study is the first to establish quantitative guidance values for iodine concentration and iodine perfusion ratios of various organs and anatomical landmarks obtained from a large cohort of patients without radiological tumor burden. While the effect of BMI can be neutralized by normalization to the iodine content of the abdominal aorta, we report on sex- and age-specific differences.

DECT-derived iodine quantification accurately depicts contrast media distribution in all technological DECT approaches and has been shown to serve as a surrogate for both organ/lesion perfusion and tumor vasculature [8, 9, 19, 28]. Over the past years, various studies investigated the usefulness of iodine quantification in clinical settings, such as vascular diseases and lesion detection and characterization [20, 21, 29, 30]. In order to evaluate the applicability of iodine maps as a quantitative parameter, particular for oncologic imaging, understanding inter-individual variability and distribution of iodinated contrast media in individual without tumor burden is crucial. So far, only few studies investigated physiological iodine concentrations in certain tissues, such as lymph nodes and myocardial tissue [26, 27]. Reference values for quantitative iodine values in parenchymatous organs are sparse. The few studies available provide absolute iodine concentrations for the liver and kidney, only. While these reports are based on observations of < 200 examinations, our findings are in line with theirs substantiating their validity [10, 19, 31].

A recent study by Sauter et al investigated iodine distribution in inguinal, mediastinal, and cervical lymph nodes using the same scanner and a comparable imaging protocol [27]. They report reference values well in line with ours, yet report a markedly higher iodine uptake for the neck compared with axillary and inguinal lymph nodes; however, they used different imaging protocols for these. While they did not report on an influence of BMI, sex, and/or age on absolute iodine concentrations, they normalized their results to the average concentration in their overall study population, which did not reduce the spread of values found [27]. Contrary, we normalized our data to the IC in the abdominal aorta for each scan and reduced any effect resulting from BMI and highlighting differences between sex and/or age. Considering these differences, subtle differences will need to be re-evaluated when used for clinical decision-making: For example, Tawfik et al report iodine values of 2.34 ± 0.45 mg/ml and 2.86 ± 0.37 for metastatic and normal lymph nodes in head and cancer; however, there also is a clear difference in age composition between both groups (60.5 and 40.0 years, respectively) [32]. While these reports are limited to lymph nodes, our data suggests that similar patterns may account for parenchymatous organs and other landmarks. Hence, care should be taken in providing cutoff values of iodine concentration to differentiate between healthy and malignant abdominal lesions. Particularly in studies with small patient samples, any bias with regard to age and/or sex should be considered and accounted, e.g., in studies with cohorts of higher age, an adaption to decreased perfusion values should be conducted.

Considering the widespread differences in iodine perfusion ratio regarding patient age and patient sex in almost all abdominal organs when applying a fixed volume of contrast media, we suggest normalizing absolute iodine concentrations to adjust for the ratio of body weight and applied amount of contrast media. Additionally, previous studies highlighted a certain intra-individual variance in patients undergoing repetitive examinations, which aggravates the definition of iodine concentration cutoff values [10, 19].

Besides its retrospective study design, there are several limitations to our study that need to be discussed. First, we only included patients that were referred to thoracoabdominal staging CT due to different types of skin cancer. Although in each case the absence of visible tumor burden was confirmed by imaging follow-up, non-visible tumor burden may still be present, and a certain selection bias regarding the patient collective cannot be ruled out. Additionally, few patients were taking specific medications at the timepoint of imaging, such as checkpoint inhibitors or interferons, which may affect contrast media distribution. Nonetheless, we consider our patient cohort as healthy as possible when investigating patients that underwent CT examinations of the chest and abdomen without radiation exposure of healthy volunteers. Second, the presented results only account for portal venous phase examinations using a similar acquisition protocol. Contrast media application protocols are topic of ongoing discussion and research. While many reasons and studies argue in favor of body weight-adapted application protocols, fixed bolus protocols are still widely used. Our results demonstrate that, when applying a fixed volume of contrast media, normalizing absolute iodine concentrations eliminates the effect of body weight on distribution. Furthermore, we solely assessed portal venous phase images as previous studies demonstrated a higher variability of iodine concentration in arterial phase examinations [19]; however, arterial phase images are often required for precise diagnosis. Third, a fixed approach of 120 kVp was used in this study, while it may be argued that obese patients should be examined with a higher tube voltage of 140 kVp. The same accounts for other acquisition and reconstruction settings: While our study followed our department’s routine, including reconstruction with a fixed matrix, different matrix sizes may impact iodine quantification. Furthermore, iodine maps were reconstructed with a soft tissue kernel, while the impact of different kernels on quantification accuracy has not been investigated. With regard to iodine concentration, comparable quantification accuracy has been demonstrated irrespective of tube voltages in SDCT by Pelgrim et al [16]. Furthermore, while all scans were screened for conditions that may compromise measurements, such as apparent perfusion deficits, a risk of bias remains. Last, our results were obtained on a dual-layer SDCT only, while an inter-vendor comparison was out of scope of this study. Yet, as different studies showed that iodine quantification can be accurately performed on all different DECT scanners [16, 18], examinations with a comparable examination protocol can be expected to provide similar results. Nevertheless, a multi-center, multi-vendor, and multi-contrast protocol study design would be required to define generally valid reference values. To render quantitative imaging parameters from (dual-energy) computed tomography clinically applicable, international agreement and guidance on image acquisition, reconstruction, and postprocessing is desirable. Such recommendations should be defined by professional societies in close cooperation with hardware and software providers. Similar efforts have been successfully made by the Quantitative Imaging Biomarkers Alliance and European Imaging Biomarkers Alliance with regard to different imaging techniques; however, quantitative maps from DECT have not found representation in these to date [33, 34].

To conclude, this study provides guidance values of iodine concentration valid for examinations with a delay of 50 s after bolus tracking obtained from a large-scale cohort of patients without radiological tumor burden. In case of fixed contrast media application, we recommend a normalization of iodine concentration to adjust for body weight and the amount of applied contrast agent. The scatter and the differences between sex and age groups we report should be considered when performing iodine measurements in scientific studies. Particularly, care should be taken in interpretation of iodine values in clinical settings.

## Abbreviations

BMI: Body mass index
CI: Conventional images
DECT: Dual-energy computed tomography
DICOM: Digital imaging and communications in medicine
IM: Iodine maps
RECIST: Response evaluation criteria in solid tumors
SDCT: Dual-layer spectral detector computed tomography
